# Isolation and genetic characterization of *Toxoplasma gondii* in Spanish sheep flocks

**DOI:** 10.1186/s13071-020-04275-z

**Published:** 2020-08-05

**Authors:** Mercedes Fernández-Escobar, Rafael Calero-Bernal, Julio Benavides, Javier Regidor-Cerrillo, María Cristina Guerrero-Molina, Daniel Gutiérrez-Expósito, Esther Collantes-Fernández, Luis Miguel Ortega-Mora

**Affiliations:** 1 SALUVET, Animal Health Department, Faculty of Veterinary Sciences, Complutense University of Madrid, Ciudad Universitaria s/n, 28040 Madrid, Spain; 2 Instituto de Ganadería de Montaña (CSIC-ULE), 24346 León, Spain; 3 SALUVET-innova S.L, Faculty of Veterinary Sciences, Complutense University of Madrid, Ciudad Universitaria s/n, 28040 Madrid, Spain

**Keywords:** *Toxoplasma gondii*, Sheep, Abortion, Isolates, Genotyping, Sequencing, Population structure, Spain

## Abstract

**Background:**

*Toxoplasma gondii* is a major cause of abortion in small ruminants and presents a zoonotic risk when undercooked meat containing cysts is consumed. The aim of the present study was to investigate the genetic diversity among the *T. gondii* strains circulating in ovine livestock in Spain.

**Methods:**

Selected samples collected from abortion outbreaks due to toxoplasmosis (*n* = 31) and from chronically infected adult sheep at slaughterhouses (*n* = 50) in different Spanish regions were bioassayed in mice, aiming at parasite isolation. In addition, all original clinical samples and the resulting isolates were genotyped by multi-nested PCR-RFLP analysis of 11 molecular markers and by PCR-DNA sequencing of portions of the *SAG3*, *GRA6* and *GRA7* genes.

**Results:**

As a result, 30 isolates were obtained from 9 Spanish regions: 10 isolates from abortion-derived samples and 20 isolates from adult myocardial tissues. Overall, 3 genotypes were found: ToxoDB#3 (type II *PRU* variant) in 90% (27/30) of isolates, ToxoDB#2 (clonal type III) in 6.7% (2/30), and ToxoDB#1 (clonal type II) in 3.3% (1/30). When *T. gondii*-positive tissue samples (*n* = 151) were directly subjected to RFLP genotyping, complete restriction profiles were obtained for 33% of samples, and up to 98% of the specimens belonged to the type II *PRU* variant. A foetal brain showed a clonal type II pattern, and four specimens showed unexpected type I alleles at the *SAG3* marker, including two foetal brains that showed I + II alleles as co-infection events. Amplicons of *SAG3*, *GRA6* and *GRA7* obtained from isolates and clinical samples were subjected to sequencing, allowing us to confirm RFLP results and to detect different single-nucleotide polymorphisms.

**Conclusions:**

The present study informed the existence of a predominant type II *PRU* variant genotype (ToxoDB#3) infecting domestic sheep in Spain, in both abortion cases and chronic infections in adults, coexisting with other clonal (ToxoDB#1 and ToxoDB#2), much less frequent genotypes, as well as polymorphic strains as revealed by clinical sample genotyping. The use of multilocus sequence typing aided in accurately estimating *T. gondii* intragenotype diversity. 
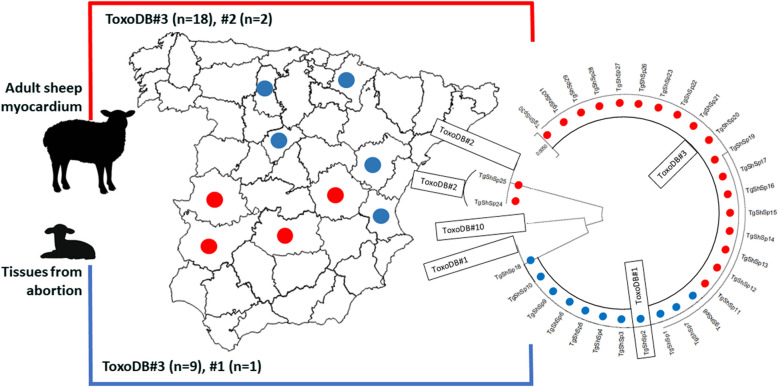

## Background

*Toxoplasma gondii* (Apicomplexa) is known as one of the main causes of ovine reproductive failure, causing significant economic losses to the sheep industry worldwide [1–3]. Among other factors, such as strain virulence and parasite stage at the time of infection [4, 5], clinical manifestations of ovine toxoplasmosis mostly depend on the pregnancy stage at which the primary infection occurs, ranging from early embryonic death with reabsorption to stillbirth or neonatal death, or even the birth of transplacentally infected lambs (congenital toxoplasmosis). In Europe, there is little information about *T. gondii* as the aetiological agent of ovine abortion outbreaks; nevertheless, similar rates of *T. gondii*-specific DNA have been detected in sheep abortion tissues submitted for diagnosis in distinct countries, i.e. in 10% of the ovine abortion-derived tissues from Ireland [6], 6–11% from UK [7], 11.1–18.1% from the Sardinia region, Italy [8, 9], 10.6% from Germany [10], and in 5.4–18.9% from Spain, as observed in previous reports [11–13].

The global seroprevalence of *T. gondii* in sheep flocks ranges between 3–98%, but the results are dependent on factors such as the age of the ewes or the management system [1]. In southern Spain, individual seroprevalence figures ranged between 41.2–49.3% in sheep flocks [14, 15], in agreement with rates found in other Mediterranean countries [3], giving the idea of a widespread prevalence.

Moreover, the role of *T. gondii* as a major pathogen in public health is well known, especially when raw or undercooked meat containing encysted bradyzoites is consumed [16, 17]. A risk assessment study estimated that the consumption of undercooked ovine meat is responsible for 14% of meat-related *T. gondii* infections in the Dutch population [18].

Vast research on *T. gondii* population structure, diversity, and geographical distribution is being conducted worldwide [19, 20]. Despite the importance of the ovine industry in Europe, information about *T. gondii* strains circulating in European ovine livestock is scarce (Table 1); while most ovine isolates and genotyping descriptions in Europe are clonal [21], some specific findings of novel genotypes [22] and non-clonal isolates [23], along with mixed infections [24], deserve attention. To date, no data are available from Spain.

**Table 1:** Summary of studies reporting *T. gondii* genotypes circulating in ruminant livestock in Europe

Country	Host species	n	Type (%)				Method	References
			I	II	III	MRA		
Isolates								
France	Sheep	8	–	100	–	–	MS	[40]
	Sheep	46	–	97.8	2.2	–	RFLP-ML + MS	[21]
	Cattle	2	–	100	–	–	MS	[68]
Italy	Sheep	5	–	–	–	100	RFLP-ML	[23]
Portugal	Cattle	1	100	–	–	–	RFLP-ML + MS	[22]
Romania	Goat	2	–	100	–	–	MS	[69]
Serbia	Sheep	1	–	100	–	–	RFLP-ML	[70]
UK	Sheep	2	–	100	–	–	RFLP-*SAG2*	[47]
Clinical samples								
Ireland	Sheep (foetal tissues)	19	–	79	21	–	RFLP-ML	[6]
Italy	Goat (milk)	10	10	–	40	50	RFLP-ML	[71]
	Sheep (placental and foetal tissues)	21	–	100	–	–	RFLP-ML	[48]
	Sheep (milk)	1	100	–	–	–	RFLP-*SAG3*	[72]
	Cattle (skeletal muscle)	6	66.6	16.6	16.6	–	RFLP-ML	[51]
	Sheep (meat)	15	–	100	–	–	*B1*-Seq	[77]
	Goat (meat)	3	–	100	–	–		
Poland	Goat (milk)	25	–	–	100	–	RFLP-ML	[73]
Portugal	Sheep (myocardium)	6	–	100	–	33.3	RFLP-*SAG2*	[74]
	Goat (myocardium)	3	–	100	–	–		
	Cattle (myocardium)	3	–	100	–	–		
The Netherlands	Sheep (myocardium)	13	–	100	–	–	MS + *GRA6*-Seq	[46]
Slovakia	Goat (milk)	14	–	100	–	–	RFLP-*SAG2*	[75]
Switzerland	Sheep (diaphragm)	5	–	–	–	100	RFLP-ML	[24]
	Cattle (diaphragm)	9				100^a^		
	Sheep (diaphragm)	5	–	40	–	60	RFLP-ML	[76]
	Cattle (diaphragm)	9	–	–	–	100^a^		
UK	Sheep (placental tissues)	13	–	100	–	–	RFLP-*SAG2*	[47]
	Sheep (meat)	6	60	–	–	40	RFLP-*SAG2*	[45]
	Cattle (meat)	1	–	–	–	100		

^a^Incomplete markers resembling atypical or recombinant patterns

MRA, patterns showing mixed infections; recombinants or atypical; MS, microsatellites; ML, multilocus; Seq, sequencing

This paper presents the genetic characterization of *T. gondii* ovine isolates and clinical samples obtained from abortion tissues and chronically infected adult animals, providing a picture of the genetic population of *T. gondii* infecting sheep in Spain.

## Methods

### Study design and sample collection

**Fig. 1:**
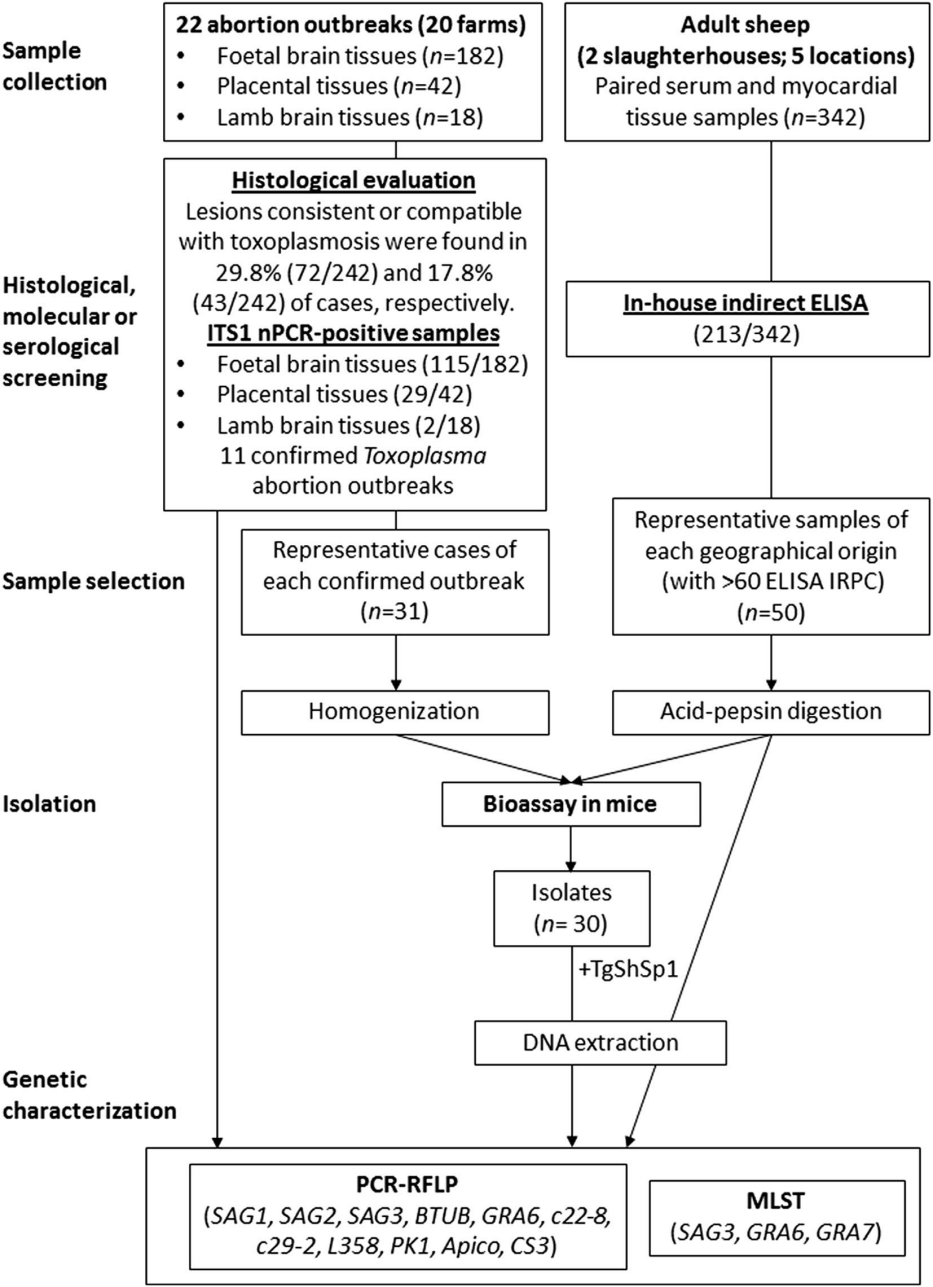
Study design

A workflow of the present study is shown in Fig. 1. Aiming to maximize the geographical extension covered within Spain and hypothesizing higher probabilities to describe genetic diversity among isolates, 2 types of tissue samples were collected for parasite isolation: (i) tissues derived from suspected *Toxoplasma*-related abortion outbreaks; and (ii) myocardial tissues from adult animals collected in authorized slaughterhouses. In this sense, between 2015 and 2018, foetal brains (*n* = 182), brains from weak lambs that died shortly after birth (*n* = 18), and placental/cotyledonary tissues (*n* = 42), were collected from 20 geographically distant farms of 22 different abortion cases (Table 2). Additionally, between February 2018 and July 2018, 342 paired serum and myocardial tissue samples were collected from adult animals slaughtered for human consumption at 2 different authorized slaughterhouses from Cáceres and Ciudad Real provinces (western and central Spain, respectively) (Table 3). The blood samples were collected with BD PLUS Serum tubes (Vacutainer; BD, Franklin Lakes, USA) at the bleeding step after the animals were euthanized, and half of the heart was taken during the evisceration process and individually stored refrigerated at 4 ℃ in labelled zip-lock plastic bags until analysis. Sampling covered a representative area within Spanish ovine farming, as samples were collected from 7 regions, representing 74.5% of the ovine census (16.6 million) in Spain [25].

**Table 2:** Epidemiological data on toxoplasmosis-like abortion outbreaks in sheep flocks in Spain (2015–2018): etiology confirmation and T. gondii isolation

Sample ID	T. gondii-associated abortion outbreak ID	Municipality (province)	Description^a^	Period of time (lambing season)	Samples collected^b^			Isolate designation (original sample)	RFLP genotype ID# (ToxoDB)
					Foetal brain tissues	Placental tissues	Lamb brain tissues		
15/121	1	Fuentes de Valdepero (Palencia, North)	Assaf, 1450, 20%	2015/2016	3 (3, 2, 1)	2 (2, 2, 0)	–	TgShSp1 (15/121.4)^c^	#3
15/141	2	Artajona (Navarra, North)	Assaf, 3800, 10%	2015/2016	2 (2, 1, 1)	–	–	TgShSp2 (15/141.2)	#1
17/4, 17, 18, 19, 21, 24, 28	3	Autillo de Campos (Palencia, North)	Assaf, 2800, 4.3%	2016/2017	24 (22, 6, 6)	6 (6, 3, 1)	7 (1, 1, 0)	TgShSp3 (17/4.1); TgShSp4 (17/21.2); TgShSp5 (17/28.1); TgShSp6 (17/19.3)	#3
17/61	–	Villaconancio (Palencia, North)	Assaf, 600, 5%, Border disease virus	2016/2017	2 (0, 0, 1)	–	–	–	
17/5, 15	4^d^	Benavente (Zamora, North West)	Assaf, 500, 10%	2016/2017	3 (2, 2, 1)	3 (2, 0, 0)	–	–	
17/6, 20, 22, 23, 25, 27, 41	5^d^	Mayorga (Valladolid, North)	Assaf, 1600, 10%	2016/2017	11 (5, 2, 2)	4 (2, 0, 2)	–	–	
17/94, 17 2/7, 17 2/6, 17 2/8, 17/29, 17/32, 17/36, 17/52 17/62	6^d^	Villamañán (León, North-West)	Assaf, NA	2016/2017	14 (6, 4, 2)	7 (3, 0, 0)	5 (0, 0, 0)	–	
17/42	–	Alcarraz (Lérida, North-East)	Lacaune, 800, 25%	2016/2017	4 (0, 0, 0)	1 (0, 0, 0)	–	–	
17/49	–	Fuentes de Valdepero (Palencia, North East)	Assaf, 1600, 5%	2016/2017	2 (0, 0, 0)	1 (0, 0)	–	–	
17/220, 221, 222, 223, 224, 225, 18/1, 4, 10	7	Navas de Oro (Segovia, Centre)	Merino, 900, 83%	2017/2018	12 (10, 5, 2)	6 (6, 1, 0)	3 (1, 1, 0)	TgShSp7 (18/4.2)	#3
17/227	–	Santa Cruz de Mudela (Ciudad Real, Centre)	Manchega, 1200, 5%	2017/2018	3 (0,0,0)	–	–	–	
18/3	–	Benavente (Zamora, North–West)	Assaf, 500, 15%, Neospora caninum	2017/2018	1 (0, 0, 0)	1 (0, 0, 0)	–	–	
18/12	–	Benegiles (Zamora, North–West)	Assaf, 1049, 15.3%, Neospora caninum	2017/2018	2 (0, 1, 0)	–	–	–	
18/5, 17	–	Casas de Juan Núñez (Albacete, East)	Manchega, 3000, 40%	2017/2018	6 (0, 0, 0)	1 (0, 0, 0)	–	–	
18/6	–	Ledesma (Salamanca, West)	Castellana, 20, 25%, Neospora caninum	2017/2018	1(0, 1, 0)	1 (0, 0, 0)	–	–	
18/7	8	Catadau (Valencia, East)	Lacaune, 2400, 21%	2017/2018	2 (2, 2, 0)		–	TgShSp8 (18/7.2)	#3
18/14, 15, 16, 18, 20	9	Cuevas de Almudén (Teruel, East)	Lacaune, 700, 50%	2017/2018	62 (50, 29, 18)	6 (6, 5, 1)	3 (0,0,0)	TgShSp9 (18/18.1); TgShSp10 (18/15.1); TgShSp18 (18/16.1)	#3
18/23	–	Moral de la Reina (Valladolid, North)	Assaf, 3000, 15%	2017/2018	5 (0, 0, 0)	1 (0, 0, 0)	–	–	
18/36, 38	–	Valdetorres (Badajoz, West)	Merino, 450, 20%, Neospora caninum	2017/2018	3 (0, 1, 1)	–	–	–	
18/21	–	Autillo de Campos (Palencia, North)	Assaf, 3000, > 1%	2017/2018	5 (0, 0, 0)	–	–	–	
18/219, 228	10^d^	Villafrechos (Valladolid, North)	Assaf, 1100, 12%	2017/2018	4 (2, 2, 0)	–	–	–	
18/222, 226	11^d^	Aguilar de Campos (Valladolid, North)	Lacaune, 2500, 25%, Coxiella burnetii	2017/2018	11 (11, 2, 4)	2 (2, 0, 0)	–	–	
Total	11				182 (115, 59, 3, 9)	42 (29, 11, 4)	18 (2, 2, 0)		

^a^Breed, flock size, % of abortion, other pathogens detected. Screening for common protozoal, bacterial and viral abortifacient agents was performed as reported in references [26] and [27]

^b^PCR-positive cases, cases with consistent lesions, cases with compatible lesions

^c^Isolation reported in reference [32]

^d^Bioassay in mice was not successful or was not carried out due to previous tissue freezing

na, data not available

**Table 3:** Summarized data on adult sheep myocardial tissue sample collection in authorized slaughterhouses in central and western Spain (2018)

Animal origin (province)	Breeding area	Age	Breed	No. of serum samples		ELISA IRPC	Isolate ID
				Samples analysed	ELISA-positive (%)		
Plasencia (Cáceres)	West	Adult (4–5 years-old)	Merino	100	39.0 (39/100)	85.0	TgShSp11
						76.9	TgShSp12
						81.1	TgShSp13
						81.6	TgShSp14
						66.3	TgShSp15
						88.1	TgShSp19
						85.1	TgShSp20
						92.0	TgShSp21
						66.0	TgShSp22
						74.7	TgShSp28
Alburquerque (Badajoz)	South–West	Adult (4–5 years-old)	Merino	100	95.0 (95/100)	107.2	TgShSp16
						115.2	TgShSp17
						113.6	TgShSp23
						86.7	TgShSp27
						122.6	TgShSp31
Sisante (Cuenca)	Centre	Adult (4–5 years-old)	Manchega × Lacaune	42	47.6 (20/42)	75.1	TgShSp26
						115.5	TgShSp30
Valdepeñas (Ciudad Real)	Centre	Adult (4–5 years-old)	Manchega × Lacaune	50	40.0 (20/50)	89.0	TgShSp24
						82.5	TgShSp25
Puertollano (Ciudad Real)	Centre	Adult (4–5 years-old)	Manchega × Lacaune	50	78.0 (39/50)	107.4	TgShSp29
Total	–	–	–	342	62.3 (213/342)	–	–

### Histological, molecular and serological diagnosis for sample selection

In abortion cases, brains from foetuses or dead lambs, and placental samples when available, were collected for histological, molecular, and mouse bioassay analyses. Initial screening for common protozoan, bacterial, and viral abortifacient agents was performed as reported elsewhere [26, 27]. Histological processing and evaluation were carried out following previous descriptions [12]. The cases were classified according to observed lesions as follows: (i) no significant lesions; (ii) lesions suggesting conditions other than toxoplasmosis; (iii) lesions compatible with toxoplasmosis (diffuse congestion and/or multifocal leukomalacia); and (iv) lesions consistent with toxoplasmosis (multifocal areas of necrosis at the placenta or glial foci with a central area of necrosis in the brain). Due to the low sensitivity and specificity of histological diagnosis, the selection of tissue samples for parasite isolation was carried out by *T. gondii* DNA detection by PCR. Genomic DNA was extracted from three different 50-mg pieces of each tissue using the Maxwell® 16 Mouse Tail DNA Purification Kit (Promega, Alcobendas, Spain), and *T. gondii* DNA detection was carried out by single-tube nested PCR amplification of the specific ITS1 region as previously described [28]. Within PCR-positive tissues, only representative cases with a lesser degree of autolysis of each confirmed outbreak were selected for the isolation assay (*n* = 31) to maximize the isolation success and geographic coverage of the study.

Regarding adult animals, *T. gondii*-specific IgG antibody levels in ovine serum samples were measured using an in-house indirect ELISA as previously described [28], considering the cut-off at 20 for ELISA IRPC (relative index per cent). Likewise, only those myocardial tissues associated with the highest antibody titres (> 60 ELISA IRPC) were selected for the mouse bioassay (*n* = 50) (Table 3).

### Bioassay in mice

Five to 15 g of brain tissue from abortion cases was suspended in a proportional volume (w/v) of PBS supplemented with penicillin (1000 IU/ml; Sigma-Aldrich, Madrid, Spain) and streptomycin (100 μg/ml; Sigma-Aldrich) [1], homogenized in a paddle blender (IUL-Maxicator, Masticator Classic 400 ml; Geneq, Quebec, Canada), centrifuged (1200×*g*, 10 min, 4 ℃), and then passed through a 20 G needle prior to subcutaneous inoculation into 2 or 3 female Swiss/CD1 mice (Janvier Labs, Laval, France) per tissue sample [29]. Additionally, hearts (portions of 50 g/each) from selected seropositive adult animals were subjected to acid-pepsin artificial digestion [1] prior to bioassay in 3 female Swiss/CD1 mice. The resulting inocula were also subjected to ITS1 nested PCR [28] to discern whether further genotyping analysis could be possible directly on these samples.

Mice were observed daily, and clinical signs were scored [30]. Tissue imprints of brains and lungs from mice that died were examined for tachyzoite or tissue cyst presence. At 30 dpi (days post-inoculation), surviving mice were bled, and serum samples were collected for anti-*T. gondii* IgG antibodies detection by an indirect fluorescent antibody test (IFAT) [31], using an anti-mouse IgG conjugated to FITC (Sigma-Aldrich) diluted 1:64 in Evans Blue (Sigma-Aldrich) and considering the cut-off at 1:25. Seropositive mice were sacrificed at 42 dpi, and a fraction of freshly recovered brain tissue was homogenized in PBS supplemented with antibiotics by passing through tapered cross-section needles (20–25 G) to be intraperitoneally (IP) inoculated into two additional female Swiss/CD1 mice. At 7 dpi, peritoneal cavity flushes were aseptically collected from mice and used for *in vitro* culture.

### *In vitro* cultivation

Peritoneal exudates of infected Swiss/CD1 mice were seeded into African green monkey kidney-derived cells (MARC-145 line) and maintained by serial passages. Cells were cultured in DMEM (Gibco, Thermo Fisher Scientific, Waltham, MA, USA) supplemented with foetal bovine serum (FBS) (Gibco), penicillin (100 U/ml), streptomycin (100 μg/ml) and amphotericin B (0.25 μg/ml) (Lonza Group, Basel, Switzerland) at 37 ℃ and 5% CO_2_ in 75 or 25 cm^2^ tissue culture flasks. Tachyzoites from successfully grown cultures were harvested from the medium for DNA isolation, and infected cells were suspended in FBS supplemented with 10% of DMSO (dimethyl sulfoxide; Sigma-Aldrich) and cryopreserved in liquid nitrogen for further studies as described previously [1].

### Genetic characterization of *T. gondii*

*Toxoplasma gondii* DNA was extracted from cell-culture-derived tachyzoites of all 30 isolates obtained, along with the isolate TgShSp1 [32]. Strain typing was performed by the widely used PCR-restriction fragment length polymorphism (RFLP) method based on *SAG1*, *SAG2* (5’-3’ *SAG2*, and alt*. SAG2*), *SAG3*, *BTUB*, *GRA6*, *c22-8*, *c29-2*, *L358*, *PK1* and *Apico* markers [33]. An additional RFLP marker, *CS3*, was included in the present study due to its proven link with the virulence of *T. gondii* strains in mice [34]. Reference strains of *T. gondii* were also incorporated in genotyping, including clonal type I (TgRH), clonal type II (TgMe49) and clonal type III (TgNED). Genotyping was also directly applied to DNA extracted from all brain and placental tissues in which *T. gondii* had been previously detected (*n* = 133) and from the *T. gondii* PCR-positive digests of sheep myocardial tissues inoculated into mice (*n* = 18). RFLP genotype numbers were assigned according to the ToxoDB database (https://toxodb.org/toxo/).

### Multilocus sequence typing (MLST) analysis

We conducted PCR sequencing of 3 polymorphic genes, *SAG3*, *GRA6* and *GRA7*, on all 31 isolates and clinical samples with previous successful nested PCR amplification of each marker (*SAG3*, *n* = 123; *GRA6*, *n* = 108; *GRA7*, *n* = 99) to provide sequence-based genotyping. Gene amplification of *SAG3* and *GRA6* resulted from the above-described Mn-PCR-RFLP method, and *GRA7* amplifications were obtained by nested PCR using specific primer pairs [35]. PCR products were sent to the Center for Genomic Technologies of the Complutense University of Madrid (Spain) for direct sequencing. Briefly, amplicons were sequenced in both directions with the same internal primer pair used for amplification employing a BigDye Terminator v3.1 Cycle Sequencing Kit (Applied Biosystems, Carlsbad, CA, USA) and a 3730 × l DNA Sequence Analyser (Applied Biosystems). Sequencing was successful for 121 out of 123 *SAG3*-PCR positives, 77 out of 108 *GRA6*-PCR positives, and all 99 *GRA7*-PCR positives. The resulting sequences were imported, read, edited manually if necessary, and analysed using BioEdit software, version 7.0.5.3 [36]. Generated DNA consensus sequences were aligned to appropriate reference sequences using MEGA X software (http://www.megasoftware.net/) [37], and compared with sequences retrieved from the National Center for Biotechnology Information (NCBI) database through the BLAST tool (http://blast.ncbi.nlm.nih.gov/Blast.cgi).

### Phylogenetic analyses

By using DNA sequence-based phylogenetic analyses, we evaluated the population structure of *T. gondii* isolates obtained; clonal reference strains (TgRH, TgMe49 and TgNED) were included for comparison. Consensus *SAG3*, *GRA6* and *GRA7* sequences from isolates and reference strains were concatenated and aligned using MEGA X software [37] to generate an unrooted phylogenetic tree. The evolutionary history was inferred using the neighbor-joining method [38]. The evolutionary distances were computed using the maximum composite likelihood method [39].

## Results

### Parasite detection and isolation

Two hundred forty-two tissue samples (182 foetal brains, 42 placentae and 18 lamb brains) from 22 suspected *Toxoplasma*-related abortion outbreaks among 20 farms distributed all over Spain were analysed by histological evaluation and nested PCR assay (Table 2). Histological lesions consistent or compatible with toxoplasmosis were found in 29.8% (72/242) and 17.8% (43/242), respectively, of the studied cases. No lesions suggesting other conditions were found, although multifocal necrotic glial foci and protozoan tissue cysts, lesions classified as characteristic of *Toxoplasma* infection, in two cases these were later confirmed to be caused by *Neospora caninum. Toxoplasma gondii*-specific DNA was detected in 60.3% (146/242) of samples; indeed, 63.2% (115/182) of foetal brains, 69.0% (29/42) of placental samples, and 11.1% (2/18) of lamb brains were positive for *T. gondii* DNA. Such findings allowed us to confirm *T. gondii* as the aetiological agent in 11 out of 22 abortion outbreaks in sheep farms (description is summarized in Table 2). Ten isolates (TgShSp2 to TgShSp10 and TgShSp18) were obtained from 31 bioassayed foetal brains (representing 5 different abortion outbreaks, Table 2).

Furthermore, *T. gondii*-specific IgG antibodies were detected in 62.3% (213/342) of adult sheep serum samples collected in slaughterhouses; 50 selected samples with the highest ELISA IRPC titres (ranging from 60.5 to 122.6; Table 3) were subjected to bioassay, and 20 isolates (TgShSp11 to 17, TgShSp19 to 31) were obtained (Table 3).

The bioassay success rate was established in 32.3% (10/31) of abortion cases and in 40% (20/50) of chronically infected adult tissues. Regarding samples from abortion outbreaks that occurred in Zamora (#4), Valladolid (# 5, #10 and #11) and León (# 6) provinces, bioassays in mice were not successful or were not carried out due to previous freezing of the tissues (Table 2).

### PCR-RFLP genotyping

Cell-culture-derived tachyzoites from all 31 isolates (including TgShSp1) were successfully typed, revealing 3 different genotypes: ToxoDB#3 (90.3%; 28/31 isolates); ToxoDB#2 (6.5%; 2/31); and ToxoDB#1 (3.2%; 1/31). Although ToxoDB#3 was the most frequently found genotype, and ToxoDB#2 was detected only in chronically infected adult animals, no specific dominance of any RFLP genotype appears to be involved in abortion cases or chronic infections (Additional file 1: Table S1). The *CS3* marker, a gene with a suggested high predictive value for virulence in mice [34], resulted in the type II allele in all isolates but 2 (TgShSp24 and 25), exhibiting a type III allele.

When PCR-RFLP assays were applied to *T. gondii* DNA-positive brain (*n* = 108) and placental tissues (*n* = 25) obtained from abortion outbreaks (Additional file 2: Table S2), and to myocardial sample digests (*n* = 18) (Additional file 3: Table S3), more complexity was observed, revealing co-infection events and suggesting the possible selection of certain strains during bioassay experiments. Amplifications yielded complete RFLP profiles for approximately 33% of specimens, with up to 98% belonging to the type II *PRU* variant (ToxoDB#3). Although incomplete RFLP profiles were obtained in some samples, allelic variations were detected in the *SAG3* marker (Additional file 2: Table S2, Additional file 3: Table S3). Infection with multiple *T. gondii* strains in the same foetus was detected in 2 brain tissues collected in outbreak #3 (Table 2), which occurred in 2017 in Palencia Province (North Spain) (ID#17/21.1 and #17/21.2), due to the coexistence of type II and type I *SAG3* alleles in the same tissue. Apart from that, a type I allele was also detected in another foetal brain tissue from the same outbreak (ID#17/28.1) and in a myocardial sample (digest) from an adult sheep from Badajoz Province (Southwest Spain) (ID: BA18 G#34).

### MLST genotyping

PCR-DNA sequencing-based genotyping considering 3 polymorphic genes, *SAG3*, *GRA6* and *GRA7*, revealed that most isolates and samples showed complete sequence homology with either TgMe49 (clonal type II) or TgNED sequences (clonal type III), supporting the RFLP results in the case of the *SAG3* and *GRA6* markers (Additional file 1: Table S1, Additional file 2: Table S2, Additional file 3: Table S3).

The *SAG3* sequence alignment of the samples (and isolates) that showed a type II allele identified a single-nucleotide polymorphism (SNP), G1691T, that divides our clonal type II (ToxoDB#1) and type II *PRU* variant (ToxoDB#3) isolates and samples into two well-defined groups. The first group had 100% homology with the TgMe49 reference sequence included and others deposited in GenBank, such as JX218226 (IIa *SAG3* allele, MT361125), and the other group (G1691T) showed 100% identity with ovine (KU599412; KU599407) or caprine (KU599396) isolate sequences deposited (IIb *SAG3* allele, MT361126) and leads to an amino acid change at codon 368 from Met to Ile. Concerning the incidence of each *SAG3* type II allele, all outbreaks described were homogeneous, presenting one allele spread over all specimens; of note, only outbreak #6 occurring in León Province during 2017 presented foetuses infected by parasites showing alleles IIa and IIb. There appears to be a higher incidence of the *SAG3* IIa allele in those outbreaks occurring in 2017, while the IIb allele seems to be more frequent in outbreaks during 2018. Regarding sample ID#17/28.1, which showed a type I allele by PCR-RFLP confirmed by PCR sequencing, a double peak was detected at position 1113 of the gene sequence, indicating a co-infection event. Between both alleles detected, one of them showed 100% homology with the TgRH reference strain sequence included and others deposited in GenBank (JX218225; AF340227) (Ia *SAG3* allele, MT358429), but the other presented a SNP (T1113C) not shared by any other sequence reported previously (Ib *SAG3* allele, MT361124) and resulted in a silent mutation. When using the BLAST tool to compare resulting consensus *SAG3* sequences with those publicly available in the GenBank database obtained from sheep or goats, it was noted that the IIb *SAG3* allele detected along part of our samples is also present in some French ovine isolates (GenBank: KU599412, KU599411 and KU599407) coexisting with IIa *SAG3* alleles (GenBank: KU599409, KU599410). The same occurred with some Ethiopian ovine and caprine isolates deposited (GenBank: KU599394, KU599396, KU599399 and KU599400), showing one allele or the other. This fact also illustrated other instances of type I and III alleles present in sheep and lamb meat samples analysed in Iraq (GenBank: MK801822- MK801830).

*GRA6* marker sequencing also sustained RFLP findings; nevertheless, a double peak at position 1013 of the gene was detected in a foetal brain tissue collected in an abortion outbreak (#9, Table 2) that occurred in 2018 in Teruel Province (East Spain) (ID#18/14.5; PCR-RFLP type II allele), indicating co-infection by two strains. Between both alleles detected, one of them presented 100% homology with the TgMe49 sequence included and others deposited in GenBank (AF239285) (IIa *GRA6* allele, MT370491), but the other one showed a SNP (C1013T) not shared by any other sequence deposited previously in that database (IIb *GRA6* allele, MT370489) and was located at the 5’ UTR fragment.

Finally, *GRA7* gene sequencing enabled us to test the sequence homology of our isolates and original clinical samples with the clonal reference strains included. Concerning the isolates obtained, the analysis showed 100% homology with the TgMe49 (clonal type II) sequence included in all cases except for the TgShSp24 and TgShSp25 sequences, which were found to be identical to the TgNED (clonal type III) sequence included. Besides, the *GRA7* sequence obtained from DNA amplified from a foetal brain collected in the above-mentioned outbreak (#9, Table 2) that occurred in Teruel Province (East Spain) (ID#18/15.21), possessed a double peak at position 2688 of the gene sequence, indicating that a co-infection was also present in this tissue. Between both alleles detected, one of them presented 100% homology with the TgMe49 sequence included and others deposited in GenBank (DQ459445) (IIa *GRA7* allele, MT361127), but the other carried a SNP (C2688T) not shared by any other sequence available (IIb *GRA7* allele, MT361128), causing an amino acid change at codon 188 from Ala to Val.

### Phylogenetic analyses

**Fig. 2:**
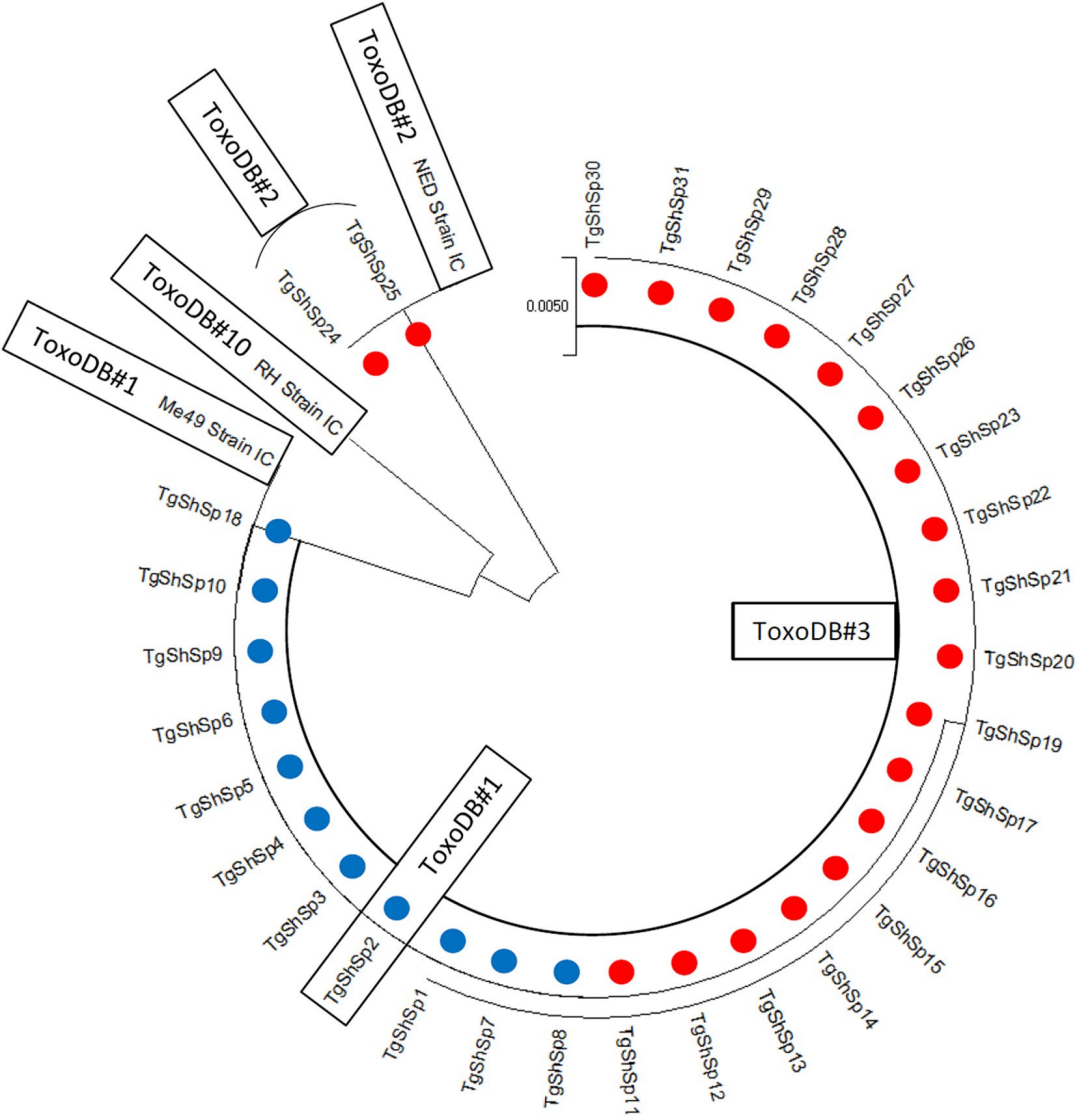
Phylogenetic tree constructed using concatenated consensus *SAG3*, *GRA6* and *GRA7* sequences of *Toxoplasma gondii* isolates obtained from abortion-derived tissues (blue dots) and myocardium from adult sheep (red dots) in Spain and T. gondii reference strains used along genotyping methods as internal controls (IC) (RH strain IC, Me49 strain IC and NED strain IC)

The population structure of *T. gondii* isolates obtained was evaluated by DNA sequence-based phylogenetic analyses. A phylogenetic tree was constructed based on concatenated *SAG3*, *GRA6* and *GRA7* sequences from isolates obtained, in addition to those from the clonal reference strains included (TgRH, TgMe49 and TgNED) (Fig. 2). Predictably, TgShSp24 and TgShSp25 isolates (type III alleles for the three markers studied) were situated next to the TgNED strain. On the other hand, the rest of the isolates (type II alleles for the three markers) formed two well-defined clusters obeying the presence of the SNP (G1691T) described previously at the *SAG3* locus.

## Discussion

*Toxoplasma gondii* has been recognized as a major cause of reproductive failure. Here, 11 toxoplasmosis-related ovine abortion outbreaks occurring in the 2015, 2016 and 2017 lambing seasons are reported. In addition to the clinical and economic interest derived from abortion outbreaks, the high seroprevalence in sheep highlights the potential public health risk posed by the consumption of lamb meat containing viable tissue cysts. In the present study, 62.3% (213/342) of blood samples collected from sheep slaughterhouses in western and central Spain were positive for *T. gondii* IgG antibodies, in agreement with the values in other European reports [3].

To the best of our knowledge, the present survey, along with two previous French investigations [21, 40], might be the only European studies comprehensive enough to analyse *T. gondii* population genetic diversity circulating through sheep flocks, and this is the first Spanish report of this nature. There is scarce knowledge regarding the genetic diversity of the *T. gondii* population in Spain. Pioneering studies were focused on genotyping human clinical samples [41] and positive tissues from wild big game species [42]; both studies corroborated a predominance of genotype II but also presented a significant prevalence of other clonal and recombinant types. To date, only two reports of *T. gondii* parasite isolation have been carried out in Spain. The first one was focused on stray cats, and typing based only on the *SAG2* locus [43] showed that 26% (*n* = 12) of isolates were type I and 74% (*n* = 34) of isolates were type II, with an absence of type III. In a second report [32], the isolate TgShSp1 (ToxoDB#3) was obtained from an ovine abortion case and was also included in the present study for further in-depth genetic analysis. We were able to isolate *T. gondii* from five additional ovine abortion outbreaks that occurred among widely geographically distributed Spanish farms during 2015–2018, as well as from chronically infected adult sheep. Overall, 30 isolates were obtained that, along with the isolate TgShSp1 [32], represented a significant cross-section of the *T. gondii* Spanish population infecting sheep, covering a wide part of the country’s territory.

Genetic characterization based on PCR-RFLP classified most isolates (90.3%; 28/31) as the type II *PRU* variant (ToxoDB#3). This fact is consistent with traditional literature referring to the predominance of *T. gondii* type II alleles among European sheep flocks [44] (summarized in Table 1). As observed in the present study, no specific genotypes have been reported in the literature in association with chronically infected adult animals (e.g. commercial meat products) [21, 40, 45, 46] or causing abortion [47, 48]. Until recently, most *T. gondii* clonal types had been recognized as infecting livestock, pets and wild animals in Europe [43, 49], but this might be biased due to the use of only *SAG2* genetic markers or a few of them for typing assays; currently, more comprehensive studies in terms of the sample size, number of molecular markers, and interactions between livestock and wildlife species have revealed an unexpectedly higher presence of polymorphic strains [50, 51], similar to direct genotyping from clinical samples.

As previously stated, in a context in which a high occurrence of *Toxoplasma* would suggest multiple exposures to the parasite during the life time of the animal [52], bioassay experiments might induce a selection of certain strains at the expense of others, resulting in an underestimation of co-infection events and, as a consequence, intraspecific diversity. Co-infection events were observed in our study in two abortion outbreaks (#3 and #9) that occurred in 2017 in Palencia Province (North Spain) and 2018 in Teruel Province (East Spain), with not only type II but also type I alleles at the *SAG3* marker detected in different foetal brain tissues (samples #17/21.1 and #17/21.2). Mixed infections have been described previously not only in ovine and porcine livestock [24, 45, 53] but also in European wildlife species [42, 54]. The type I allele at the *SAG3* marker was also detected alone in another foetal brain tissue (ID#17/28.1) from outbreak #3 and in the myocardium from an adult animal (BA18 G#34) bred in Badajoz Province (Southwest Spain), calling attention to the extension of this type I allele through livestock, as in other European studies [23, 45, 51]. Considering that the bioassay of samples #17/21.2 (Ia and IIa alleles detected) and #17/28.1 (Ia and Ib alleles present) resulted in TgShSp4 and TgShSp5 isolation (only IIa allele found), a selection of certain strains is evident during isolation experiments. It should be noted that the greatest genetic variability was detected in abortion outbreaks #3 and #9, coinciding with those from which more samples were collected, demonstrating that sampling effort is an important factor.

Phylogenetic analyses of strongly variable loci coding for virulence factors such as surface and secretory antigens, often under significant selective pressure, have been widely used to infer possible genetic population structure models, evolutionary relationships between *T. gondii* populations, reservoirs, and transmission patterns, among other factors [20, 55, 56]. Our results suggest that Spanish and French *T. gondii* populations could be genetically related based on limited *SAG3* sequences of sheep origin deposited in the GenBank database. Both sets of sequences clustered in two groups determined by the specific SNP (G1691T) described here. This may suggest common evolutionary forces or most likely common origins in livestock from both countries [56] due to a historical and intense trade exchange of sheep from Spain to France and *vice versa*. Sequences from two Ethiopian goat isolates deposited in GenBank also presented such dichotomy, possibly implying a further extension of the mutation.

The *CS3* gene has been described previously as a marker highly predictive of *T. gondii* isolates mortality in mice [34]. Bioassay results suggest a low degree of virulence for isolates obtained here, since none of the mice infected during the isolation process presented acute symptoms or died of toxoplasmosis. Our *CS3* typing results disagree with those of previous studies carried out with Brazilian and Chinese isolates of different host origins that report high mortality rates (normally above 80%) associated with type I or II alleles for the *CS3* gene and low (3.7–9.3%) or null rates with type III alleles [34, 57–60]. Contradictory results were already exposed within avirulent Brazilian isolates presenting type I [61] or type II [62] alleles for the *CS3* locus. Thus, the fact that all strains included in the studies mentioned above are polymorphic, none of them with a European or North American origin (“clonal” regions), suggests the need for further investigations to unravel the role of the gene in *Toxoplasma* virulence and clear differences between distant biogeographical global areas. Considering the known proximity of the *CS3* gene to demonstrated virulence factors such as *ROP18* and *ROP5* in the *Toxoplasma* genome (chromosome VIIa) [63–67], a linked expression with still unknown implications might be possible; therefore, research on the expression of both factors would be relevant in future studies of isolates characterization.

In conclusion, our results show that a large majority of isolates circulating around sheep farms fall within three genotypes (ToxoDB#3, 2 and 1), with some infrequent SNPs, in agreement with low genetic variability in Europe. The differential clinical outcomes observed in abortion cases draw attention to the necessity of analysing the genetic and phenotypic diversity among *Toxoplasma* parasites in Europe, especially aiming to (i) predict epidemiological changes, (ii) identify virulence factors, and (iii) design effective vaccines against field strains. Thus, increasing the effort in isolation and genotyping will provide interesting information on the epidemiology of *T. gondii* and the paradigm of One Health parasites infecting humans, livestock, and wildlife in Europe.

## Conclusions

To the best of our knowledge, the present survey constitutes the first study aiming to describe the genetic population of *T. gondii* circulating in sheep flocks in Spain. Genetic characterization of 31 strains isolated from abortion cases and chronically infected adult animals showed low genetic variability, with a predominant type II *PRU* variant genotype (ToxoDB#3) coexisting with other clonal (ToxoDB#2 and #1), much less frequent genotypes. Furthermore, when directly examining the clinical samples and inocula, the genetic richness increases, allowing the identification of other genetic variants. The present results support the hypothesis of the existence of polymorphic and overlapping strains within ovine livestock in Spain and point out the necessity of increased genotyping and sampling efforts to accurately estimate *T. gondii* intraspecific genetic diversity.

## Supplementary information

**Additional file 1: Table S1.** Genotyping allele profile obtained by PCR-RFLP and PCR-sequencing on *T. gondii* isolates.

**Additional file 2: Table S2.** Genotyping allele profile obtained by PCR-RFLP and PCR-sequencing on *T. gondii* DNA-positive clinical samples collected from abortion outbreaks.

**Additional file 3: Table S3.** Genotyping allele profile obtained by PCR-RFLP and PCR-sequencing on *T. gondii* DNA-positive adult sheep myocardium digests.

## Data Availability

Data supporting the conclusions of this article are included within the article and its Additional files 1, 2, 3. The sequences generated in the present study were submitted to the GenBank database under the following accession numbers: *SAG3* sequences (MT358429, MT361124-MT361126); *GRA6* sequences (MT370489, MT370491); and *GRA7* sequences (MT361127, MT361128). Histological samples are available from the authors upon reasonable request.

## Abbreviations

DMEM: Dulbecco’s modified Eagle’s medium
DMSO: dimethyl sulfoxide
dpi: days post-inoculation
ELISA: enzyme-linked immunosorbent assay
FBS: fetal bovine serum
FITC: fluorescein isothiocyanate
IFAT: indirect fluorescent antibody test
IP: intraperitoneally
IRPC: relative index per cent
MLST: multilocus sequence typing
PBS: phosphate-buffered saline
PCR-RFLP: polymerase chain reaction-restriction fragment length polymorphism
SNP: single nucleotide polymorphisms
