# Correction to: Isolation and genetic characterization of *Toxoplasma gondii* in Spanish sheep flocks

**DOI:** 10.1186/s13071-020-04423-5

**Published:** 2020-11-03

**Authors:** Mercedes Fernández-Escobar, Rafael Calero-Bernal, Julio Benavides, Javier Regidor-Cerrillo, María Cristina Guerrero-Molina, Daniel Gutiérrez-Expósito, Esther Collantes-Fernández, Luis Miguel Ortega-Mora

**Affiliations:** 1 SALUVET, Animal Health Department, Faculty of Veterinary Sciences, Complutense University of Madrid, Ciudad Universitaria s/n, 28040 Madrid, Spain; 2 Instituto de Ganadería de Montaña (CSIC-ULE), 24346 León, Spain; 3 SALUVET-innova S.L., Faculty of Veterinary Sciences, Complutense University of Madrid, Ciudad Universitaria s/n, 28040 Madrid, Spain

## Correction to: Parasites & Vectors (2020) 13:396 10.1186/s13071-020-04275-z

Following the publication of the original article [1] the authors flagged that, unfortunately, there are errors in Table 3 and Additional file 1: Table S1.

**Table 3:** Summarized data on adult sheep myocardial tissue sample collection in authorized slaughterhouses in central and western Spain (2018)

Animal origin (province)	Breeding area	Age	Breed	No. of serum samples		ELISA IRPC	Isolate ID
				Samples analysed	ELISA-positive (%)		
Plasencia (Cáceres)	West	Adult (4–5 years-old)	Merino	100	39.0 (39/100)	85.0	TgShSp11
						76.9	TgShSp12
						81.1	TgShSp13
						81.6	TgShSp14
						66.3	TgShSp15
						88.1	TgShSp19
						85.1	TgShSp20
						92.0	TgShSp21
						66.0	TgShSp22
						74.7	TgShSp28
Alburquerque (Badajoz)	South-West	Adult (4–5 years-old)	Merino	100	95.0 (95/100)	107.2	TgShSp16
						115.2	TgShSp17
						113.6	TgShSp23
						115.5	TgShSp30
						122.6	TgShSp31
Sisante (Cuenca)	Centre	Adult (4–5 years-old)	Manchega × Lacaune	42	47.6 (20/42)	75.1	TgShSp26
						83	TgShSp27
Valdepeñas (Ciudad Real)	Centre	Adult (4–5 years-old)	Manchega × Lacaune	50	40.0 (20/50)	89.0	TgShSp24
						82.5	TgShSp25
Puertollano (Ciudad Real)	Centre	Adult (4–5 years-old)	Manchega × Lacaune	50	78.0 (39/50)	107.4	TgShSp29
Total	–	–	–	342	62.3 (213/342)	–	–

In Table 3, the animal origin (province) of the isolate TgShSp30 was listed as Sisante (Cuenca); however, this isolate originates from Alburquerque (Badajoz). Likewise, the animal origin (province) of the isolate TgShSp27 was listed as Alburquerque (Badajoz); however, this isolate originates from Sisante (Cuenca). Additionally, the ELISA IRCP value for the serum sample of the animal from which isolate TgShSp27 was obtained is 83 instead of 86.7.

In Additional file 1: Table S1, the location of origin of the isolates TgShSp27 and TgShSp30 was mistaken in the same way. In addition, the proportion of no. infected mice/no. inoculated mice, should be changed from 2/3 to 1/3 in the case of TgShSp30, and from 1/3 to 2/3 in the case of TgShSp27 isolate.

The corrected versions of Table 3 and Additional file 1: Table S1 are provided in this correction. The authors apologise for the inconvenience caused.

## Supplementary information

**Additional file 1: Table S1.** Genotyping allele profile obtained by PCR-RFLP and PCR-sequencing on *T. gondii* isolates.
