# Supplementary material for: Isolation and genetic characterization of Toxoplasma gondii in Spanish sheep flocks
Source: Parasit Vectors. 2020 Aug 5;13:396. doi: 10.1186/s13071-020-04275-z (PMC7404076; doi:10.1186/s13071-020-04275-z)
Supplement: Supplementary file 1 — Additional file 1: Table S1. Genotyping allele profile obtained by PCR-RFLP and PCR-sequencing on T. gondii isolates. [file 13071_2020_4275_MOESM1_ESM.docx]

**Additional file 1: Table S1.** Genotyping allele profile obtained by PCR-RFLP and PCR-sequencing on *T. gondii* isolates.

|  |  | |  |  | | **PCR-RFLP alleles^a^** | | | | | | | | | | | | |  | | **PCR-Seq alleles** | | |  |
| --- | --- | --- | --- | --- | --- | --- | --- | --- | --- | --- | --- | --- | --- | --- | --- | --- | --- | --- | --- | --- | --- | --- | --- | --- |
| **ID# Isolate** | **Sample, host (location) abortion outbreak #** | **Mice bioassay (no. infected/no. inoculated)** | | | **SAG1** | **3’-SAG2** | **5’-SAG2** | **Alt. SAG2** | | **SAG3** | **BTUB** | **GRA6** | **c22-8** | **C29-2** | **L358** | **PK1** | **Apico** | **CS3** |  | **SAG3^b^** | | **GRA6^c^** | **GRA7^d^** | |
| RH | CNS, human (EEUU) | - | | | I | I/III | I/II | | I | I | I | I | I | I | I | I | I | - |  | I | | I | I | |
| Me-49 | Muscle, ovine (EEUU) | - | | | II/III | II | I/II | | II | II | II | II | II | II | II | II | II | - |  | IIa | | IIa | IIa | |
| NED | Placental tissues, human (France) | - | | | II/III | I/III | III | | III | III | III | III | III | III | III | III | III | - |  | III | | III | III | |
| TgShSp1 | Foetal brain, ovine (Palencia, Spain)  #1 | (1/1) | | | II/III | II | I/II | | II | II | II | II | II | II | II | II | I | II |  | IIb | | IIa | IIa | |
| TgShSp2 | Foetal brain, ovine (Navarra, Spain)  #2 | (2/3) | | | II/III | II | I/II | | II | II | II | II | II | II | II | II | II | II |  | IIa | | IIa | IIa | |
| TgShSp3-6 | Foetal brain, ovine (Palencia, Spain)  #3 | (1/2); (2/2); 1/2); (1/2) | | | II/III | II | I/II | | II | II | II | II | II | II | II | II | I | II |  | IIa | | IIa | IIa | |
| TgShSp7 | Foetal brain, ovine (Segovia, Spain)  #7 | (3/3) | | | II/III | II | I/II | | II | II | II | II | II | II | II | II | I | II |  | IIb | | IIa | IIa | |
| TgShSp8 | Foetal brain, ovine (Valencia, Spain)  #8 | (2/3) | | | II/III | II | I/II | | II | II | II | II | II | II | II | II | I | II |  | IIb | | IIa | IIa | |
| TgShSp9, 10, 18 | Foetal brain, ovine (Teruel, Spain)  #9 | (3/3); (3/3); (1/3) | | | II/III | II | I/II | | II | II | II | II | II | II | II | II | I | II |  | IIa | | IIa | IIa | |
| TgShSp11, 14,15,19,20,21, 28 | Myocardium, ovine (Cáceres, Spain) | (3/3);(3/3); (3/3); (2/3); (3/3); (2/3); (1/3) | | | II/III | II | I/II | | II | II | II | II | II | II | II | II | I | II |  | IIb | | IIa | IIa | |
| TgShSp12, 13, 22 | Myocardium, ovine (Cáceres, Spain) | (3/3);(3/3); (1/3) | | | II/III | II | I/II | | II | II | II | II | II | II | II | II | I | II |  | IIb | | IIa | IIa | |
| TgShSp16, 17, 23, 27, 31 | Myocardium, ovine (Badajoz, Spain) | (2/3);(3/3); (3/3); (2/3); (1/3) | | | II/III | II | I/II | | II | II | II | II | II | II | II | II | I | II |  | IIb | | IIa | IIa | |
| TgShSp24, 25 | Myocardium, ovine (Ciudad Real, Spain) | (1/3); (1/3) | | | II/III | I/III | III | | III | III | III | III | III | III | III | III | III | III |  | III | | III | III | |
| TgShSp26, 30 | Myocardium, ovine (Cuenca, Spain) | (1/3); (1/3) | | | II/III | II | I/II | | II | II | II | II | II | II | II | II | I | II |  | IIb | | IIa | IIa | |
| TgShSp29 | Myocardium, ovine (Ciudad Real, Spain) | (3/3) | | | II/III | II | I/II | | II | II | II | II | II | II | II | II | I | II |  | IIb | | IIa | IIa | |

^a^I, II or III refers to the archetypal alleles from a Type I, II or III, for each molecular marker [33].

^b^I=100% homology with GenBank accession no. AF340227 sequence; IIa=100% homology with GenBank accession no. JX218226 or MT361125 sequences; IIb=G1691T, GenBank accession no. MT361126; III=100% homology with GenBank accession no. LN714502 sequence.

^c^I=100% homology with GenBank accession no. AF239283 sequence; IIa=100% homology with GenBank accession no. AF239285 or MT370491 sequences; III=100% homology with GenBank accession no. AF239286 sequence.

^d^I=100% homology with GenBank accession no. DQ459443 sequence; IIa=100% homology with GenBank accession no. DQ459445 or MT361127 sequence; III=100% homology with GenBank accession no. DQ459455 sequence.
