# Supplementary material for: Isolation and genetic characterization of Toxoplasma gondii in Spanish sheep flocks
Source: Parasit Vectors. 2020 Aug 5;13:396. doi: 10.1186/s13071-020-04275-z (PMC7404076; doi:10.1186/s13071-020-04275-z)
Supplement: Supplementary file 2 — Additional file 2: Table S2. Genotyping allele profile obtained by PCR-RFLP and PCR-sequencing on T. gondii DNA-positive clinical samples collected from abortion outbreaks. [file 13071_2020_4275_MOESM2_ESM.docx]

**Additional file 2: Table S2.** Genotyping allele profile obtained by PCR-RFLP and PCR-sequencing on *T. gondii* DNA positive clinical samples collected from abortion outbreaks.

|  |  | **PCR-RFLP alleles^a^** | | | | | | | | | | | | |  | **PCR-Seq alleles** | | |
| --- | --- | --- | --- | --- | --- | --- | --- | --- | --- | --- | --- | --- | --- | --- | --- | --- | --- | --- |
| **ID# Sample** | **Location,**  **year,**  **abortion outbreak #** | **SAG1** | **3’-SAG2** | **5’-SAG2** | **Alt. SAG2** | **SAG3** | **BTUB** | **GRA6** | **c22-8** | **C29-2** | **L358** | **PK1** | **Apico** | **CS3** |  | **SAG3^b^** | **GRA6^c^** | **GRA7^d^** |
| **15/121.1** | Fuentes de Valdepero (Palencia)  2015  #1 | II/III | II | I/II | II | II | II | II | II | II | II | II | I | II |  | IIb | IIa | IIa |
| **15/121.2** |  | - | - | - | - | - | - | - | - | - | - | - | - | **-** |  | - | - | IIa |
| **15/121.3** |  | II/III | II | I/II | II | II | II | II | II | II | II | II | I | II |  | IIb | - | - |
| **15/121.4** |  | - | - | - | - | II | - | - | - | - | - | II | - | - |  | IIb | - | - |
| **15/121.5** |  | II/III | II | I/II | II | II | - | II | II | II | - | II | I | II |  | IIb | - | IIa |
| **15/141** | Artajona (Navarra)  2015  #2 | II/III | II | II | II | II | II | II | II | II | II | II | II | II |  | IIa | IIa | IIa |
| **17/4.1a** | Autillo de Campos (Palencia)  2017  #3 | II/III | II | I/II | - | II | II | II | II | II | II | II | I | II |  | IIa | IIa | IIa |
| **17/4.1b** |  | II/III | II | I/II | II | II | II | II | II | II | II | - | I | II |  | IIa | IIa |  |
| **17/4.2** |  | II/III | II | I/II | II | II | - | II | II | II | II | - | I | II |  | IIa | IIa | IIa |
| **17/4.3** |  | II/III | II | I/II | - | II | II | II | - | - | II | - | I | II |  | IIa | IIa | - |
| **17/4.5** |  | II/III | II | I/II | II | II | II | II | II | II | II | II | I | II |  | IIa | - | IIa |
| **17/4.6** |  | II/III | II | I/II | II | II | II | - | II | II | - | II | I | - |  | IIa | - | IIa |
| **17/17.1** |  | II/III | II | I/II | - | II | II | - | II | - | - | - | I | **-** |  | IIa | - | - |
| **17/17.2** |  | II/III | II | I/II | - | II | - | - | II | II | II | - | I | **-** |  | IIa | - | IIa |
| **17/18.1** |  | II/III | II | I/II | - | II | - | II | - | - | - | - | I | II |  | IIa | - | IIa |
| **17/18.2** |  | II/III | II | I/II | II | II | - | II | II | II | II | II | I | II |  | IIa | - | - |
| **17/18.2Pla** |  | II/III | II | I/II | II | II | II | II | II | II | II | II | I | II |  | IIa | IIa | IIa |
| **17/19.1** |  | II/III | II | I/II | - | II | - | II | - | - | - | - | I | II |  | IIa | - | - |
| **17/19.1Pla** |  | II/III | II | I/II | II | II | II | II | II | II | II | II | I | II |  | IIa | IIa | IIa |
| **17/19.2** |  | II/III | II | I/II | - | - | - | - | II | - | II | - | I | II |  | - | - | - |
| **17/19.3** |  | - | - | - | - | II | II | II | II | II | II | II | I | - |  | IIa | - | IIa |
| **17/19.3Pla** |  | II/III | II | I/II | II | II | II | II | II | II | II | II | I | II |  | IIa | IIa | IIa |
| **17/21.1** |  | II/III | II | I/II | II | I+II | II | II | - | II | II | II | I | II |  | Ia+IIa | - | IIa |
| **17/21.2** |  | - | II | I/II | II | I+II | - | II | II | II | II | II | I | - |  | Ia+IIa | IIa | IIa |
| **17/21.3** |  | II/III | II | I/II | II | II | II | II | II | II | II | II | I | II |  | IIa | - | IIa |
| **17/21.1Pla** |  | II/III | II | I/II | II | II | II | II | II | II | II | II | I | II |  | IIa | IIa | IIa |
| **17/24.1** |  | II/III | II | I/II | II | II | II | II | II | II | II | II | I | II |  | IIa | IIa | - |
| **17/24.2** |  | II/III | II | I/II | II | II | II | II | II | II | II | II | I | II |  | IIa | IIa | IIa |
| **17/24.3** |  | II/III | II | I/II | - | II | II | II | - | II | - | II | I | II |  | IIa | - | - |
| **17/28.1** |  | - | II | I/II | II | I | II | II | - | - | II | - | I | II |  | Ia+Ib | - | - |
| **17/28.2** |  | II/III | II | I/II | - | II | II | II | - | - | - | - | I | II |  | IIa | - | - |
| **17/15.1** | Benavente (Zamora)  2017  #4 | II/III | II | I/II | - | II | - | - | II | - | - | - | I | II |  | IIa | - | IIa |
| **17/15.1Pla** |  | II/III | II | I/II | II | II | II | II | II | II | II | II | I | II |  | IIa | - | - |
| **17/20.1** | Mayorga (Valladolid) 2017  #5 | II/III | II | I/II | - | II | - | - | - | - | - | - | I | II |  | IIa | - | - |
| **17/20.1Pla** |  | II/III | II | I/II | - | II | II | - | - | - | II | - | I | - |  | IIa | - | IIa |
| **17/20.2** |  | II/III | II | I/II | - | II | - | II | - | - | - | - | I | II |  | IIa | - | - |
| **17/24.4** |  | II/III | II | I/II | II | II | II | II | II | II | II | II | I | II |  | IIa | - | IIa |
| **17/24.4Pla** |  | - | II | I/II | - | - | - | - | - | II | - | II | I | - |  | - | - | - |
| **17/29.1** | Villamañán (León)  2017  #6 | II/III | II | I/II | - | II | - | - | II | - | II | - | I | II |  | IIb | - | - |
| **17/29.1Pla** |  | II/III | II | I/II | II | II | II | II | II | II | II | II | I | II |  | IIb | IIa | IIa |
| **17/32.2** |  | II/III | II | I/II | - | II | II | - | II | - | - | - | I | II |  | IIa | - | - |
| **17/33** |  | II/III | II | I/II | - | II | - | - | - | II | II | - | I | - |  | - | - | - |
| **17/33Pla** |  | II/III | II | I/II | II | II | II | II | II | II | II | II | I | II |  | IIb | - | IIa |
| **17/220.1** | Navas de Oro (Segovia) 2017/18  #7 | II/III | II | II | II | II | II | II | II | II | II | II | - | II |  | IIb | IIa | IIa |
| **17/221.1** |  | II/III | II | II | II | II | II | II | II | II | II | II | I | II |  | IIb | IIa | IIa |
| **17/222.1** |  | II/III | II | - | - | II | - | II | II | II | II | II | I | II |  | IIb | IIa | IIa |
| **17/220.2** |  | II/III | II | II | II | II | II | II | II | II | II | II | I | II |  | IIb | IIa | IIa |
| **17/221.2** |  | II/III | II | II | II | II | II | II | II | II | II | II | I | II |  | IIb | IIa | IIa |
| **17/222.2** |  | II/III | II | II | II | II | II | II | II | II | II | II | I | II |  | IIb | IIa | IIa |
| **17/223.1** |  | II/III | II | II | II | II | II | II | II | II | II | II | I | II |  | IIb | IIa | IIa |
| **17/224.1** |  | II/III | II | II | II | II | II | II | II | II | II | II | I | II |  | IIb | IIa | IIa |
| **17/225.1** |  | - | - | II | II | II | II | II | II | - | II | - | I | II |  | IIb | - | IIa |
| **17/224.2** |  | II/III | II | II | II | II | II | II | II | II | II | II | I | II |  | IIb | IIa | IIa |
| **18/4.1** |  | - | II | - | - | - | - | II | - | II | - | - | I | II |  | - | IIa | - |
| **18/4.2** |  | II/III | II | II | II | II | II | II | II | II | II | II | - | II |  | IIb | IIa | IIa |
| **18/4.3** |  | II/III | II | - | - | II | - | II | - | - | II | II | I | - |  | IIb | IIa | IIa |
| **18/4.4** |  | - | - | II | II | - | - | II | II | - | II | II | I | II |  | - | - | IIa |
| **18/4.5** |  | II/III | - | II | II | II | II | II | II | II | II | II | I | II |  | IIb | IIa | IIa |
| **18/4.8** |  | - | - | II | II | II | II | II | II | - | II | - | I | II |  | IIb | - | - |
| **18/10.C2** |  | II/III | II | II | II | II | II | II | II | II | II | II | I | II |  | IIb | IIa | IIa |
| **18/7.1** | Catadau (Valencia)  2018  #8 | II/III | II | II | II | II | II | II | II | II | II | II | I | II |  | IIb | IIa | IIa |
| **18/7.2** |  | - | II | II | II | II | - | II | II | II | II | II | I | - |  | IIb | IIa | IIa |
| **18/14.1** | Cuevas de Almudén (Teruel)  2018  #9 | - | II | - | - | II | - | - | II | - | II | II | I | II |  | IIa | - | IIa |
| **18/14.2** |  | II/III | II | II | II | II | II | II | II | II | II | II | I | II |  | IIa | IIa | IIa |
| **18/14.3** |  | II/III | II | II | II | II | - | II | - | II | II | II | I | II |  | IIa | IIa | IIa |
| **18/14.4** |  | II/III | II | II | II | II | II | II | II | II | II | II | I | II |  | IIa | IIa | IIa |
| **18/14.5** |  | - | - | II | II | II | II | II | II | II | II | II | I | II |  | IIa | IIa+IIb | IIa |
| **18/14.6** |  | II/III | II | II | II | II | II | II | II | II | II | II | - | II |  | IIa | IIa | IIa |
| **18/14.7** |  | - | - | - | - | - | - | - | - | II | - | - | I | - |  | - | - | - |
| **18/14.8** |  | II/III | II | II | II | II | II | II | II | II | II | II | I | II |  | IIa | IIa | IIa |
| **18/14.9** |  | **-** | - | - | - | - | II | II | - | - | - | - | - | - |  | - | IIa | - |
| **18/14.10** |  | II/III | II | - | - | II | - | - | - | - | - | - | I | - |  | IIa | - | IIa |
| **18/14.11** |  | II/III | II | - | - | II | II | II | II | II | II | - | I | II |  | IIa | - | IIa |
| **18/14.12** |  | II/III | II | II | II | II | II | II | II | II | II | II | I | II |  | IIa | IIa | IIa |
| **18/14.13** |  | **-** | - | II | II | II | II | II | II | II | II | II | I | II |  | IIa | IIa | IIa |
| **18/14.14** |  | **-** | - | - | - | - | - | - | II | - | - | - | I | - |  | - | - | - |
| **18/14.15** |  | II/III | II | II | II | II | II | II | II | II | II | II | I | II |  | IIa | IIa | IIa |
| **18/14.16** |  | II/III | II | - | II | - | II | II | - | II | II | II | I | II |  | - | - | - |
| **18/14.17** |  | **-** | - | - | - | II | - | - | - | - | - | - | - | - |  | IIa | - | - |
| **18/14.18** |  | **-** | - | - | - | II | - | - | - | - | - | - | - | - |  | IIa | - | - |
| **18/14.19** |  | **-** | - | - | - | II | - | - | - | II | - | - | - | II |  | IIa | - | IIa |
| **18/14.20** |  | II/III | - | I/II | II | - | II | II | - | - | II | - | I | - |  | - | - | IIa |
| **18/14.21** |  | **-** | - | - | - | II | II | - | - | - | II | - | I | II |  | IIa | - | IIa |
| **18/14.22** |  | II/III | II | II | II | II | II | - | II | - | - | II | I | - |  | IIa | - | IIa |
| **18/15.1** |  | II/III | II | II | II | II | II | II | II | II | II | II | I | II |  | IIa | IIa | IIa |
| **18/15.2** |  | II/III | II | II | II | II | II | II | II | II | II | II | I | II |  | IIa | IIa | IIa |
| **18/15.3** |  | II/III | II | II | II | II | II | II | II | II | II | II | I | II |  | IIa | IIa | IIa |
| **18/15.4** |  | II/III | II | II | II | II | II | II | II | II | II | II | - | II |  | IIa | IIa | IIa |
| **18/15.5** |  | **-** | **-** | **-** | **-** | **-** | **-** | II | II | II | **-** | **-** | I | **-** |  | - | - | - |
| **18/15.6** |  | II/III | - | II | II | II | II | II | II | II | II | - | I | II |  | IIa | - | - |
| **18/15.7** |  | II/III | II | II | II | II | II | - | II | II | II | II | I | II |  | IIa | - | IIa |
| **18/15.8** |  | II/III | II | II | II | II | II | II | II | II | II | II | I | II |  | IIa | IIa | IIa |
| **18/15.9** |  | II/III | II | II | II | II | II | II | II | II | II | II | I | II |  | IIa | IIa | IIa |
| **18/15.10** |  | II/III | II | II | II | II | II | II | II | II | II | II | I | II |  | IIa | - | IIa |
| **18/15.11** |  | **-** | II | **-** | **-** | II | - | II | - | II | II | - | I | **-** |  | IIa | - | - |
| **18/15.12** |  | II/III | II | II | II | II | II | II | II | II | II | II | I | II |  | IIa | IIa | IIa |
| **18/15.13** |  | II/III | II | II | II | II | II | II | II | II | II | II | I | II |  | IIa | IIa | IIa |
| **18/15.14** |  | II/III | **-** | II | II | II | II | II | - | II | II | - | I | II |  | IIa | IIa | IIa |
| **18/15.16** |  | II/III | II | II | II | II | II | II | II | II | II | II | I | - |  | IIa | IIa | IIa |
| **18/15.18** |  | II/III | II | II | II | II | II | II | II | II | II | II | - | II |  | IIa | IIa | IIa |
| **18/15.19** |  | II/III | **-** | **-** | **-** | **-** | **-** | II | II | - | **-** | **-** | I | II |  | - | - | IIa |
| **18/15.20** |  | II/III | II | II | II | II | II | II | II | II | II | II | - | II |  | IIa | - | IIa |
| **18/15.21** |  | **-** | II | II | II | II | II | II | - | II | II | II | - | - |  | IIa | IIa | IIa+IIb |
| **18/15.23** |  | **-** | - | II | II | II | II | - | II | - | II | - | I | - |  | IIa | - | - |
| **18/15.24** |  | **-** | II | II | II | II | - | - | II | - | - | - | I | II |  | IIa | - | - |
| **18/16.1** |  | II/III | II | II | II | II | II | II | II | II | II | II | I | II |  | IIa | IIa | IIa |
| **18/16.2** |  | **-** | **-** | **-** | **-** | II | **-** | II | - | - | **-** | **-** | I | II |  | IIa | IIa | - |
| **18/18.1** |  | II/III | II | II | II | II | II | II | II | II | II | II | I | II |  | IIa | IIa | IIa |
| **18/18.3** |  | II/III | II | II | II | II | II | II | II | II | II | II | I | II |  | IIa | IIa | IIa |
| **18/18.5** |  | II/III | II | II | II | II | II | II | II | II | II | II | I | II |  | IIa | IIa | IIa |
| **18/18.6** |  | II/III | II | II | II | II | II | II | II | II | II | II | I | II |  | IIa | IIa | IIa |
| **18/18.7** |  | II/III | II | II | II | II | II | II | II | II | II | - | I | II |  | IIa | IIa | IIa |
| **18/18.8** |  | II/III | - | - | - | - | II | II | II | II | II | II | I | II |  | - | IIa | - |
| **18/18.10** |  | II/III | II | II | II | II | II | II | II | II | II | II | - | II |  | IIa | IIa | IIa |
| **18/18.12** |  | **-** | II | II | II | II | II | II | II | II | II | II | I | II |  | IIa | IIa | IIa |
| **18/18.14** |  | II/III | II | - | - | II | II | II | II | II | II | II | I | II |  | IIa | IIa | IIa |
| **18/18.15** |  | II/III | II | II | II | II | II | II | II | II | II | II | I | II |  | IIa | - | IIa |
| **18/18.17** |  | **-** | II | - | **-** | II | - | II | - | - | II | - | I | **-** |  | IIa | - | - |
| **18/228.1** | Villafrechos (Valladolid)  2018  #10 | II/III | II | II | II | II | II | II | II | II | II | II | I | II |  | IIb | IIa | IIa |
| **18/228.2** |  | - | - | - | - | II | II | - | - | - | II | - | I | II |  | IIb | - | - |
| **18/226.1** | Aguilar de Campos (Valladolid)  2018  #11 | II/III | II | - | - | II | II | II | II | II | II | - | I | II |  | IIb | IIa | IIa |
| **18/226.2** |  | II/III | II | II | II | II | II | II | II | II | II | II | I | II |  | IIb | IIa | IIa |
| **18/226.3** |  | - | II | II | II | II | II | II | II | II | II | II | I | II |  | IIb | IIa | IIa |
| **18/226.4** |  | II/III | II | II | II | II | II | II | II | II | II | - | I | II |  | IIb | IIa | IIa |
| **18/226.5** |  | II/III | II | II | II | II | II | II | II | II | II | - | I | II |  | IIb | IIa | IIa |
| **18/226.6** |  | II/III | II | II | II | II | II | II | II | II | II | II | I | II |  | IIb | IIa | IIa |
| **18/226.7** |  | II/III | II | - | - | - | II | II | II | - | - | II | I | II |  | - | IIa | IIa |
| **18/226.8** |  | II/III | II | II | II | - | II | - | II | II | - | II | I | II |  | - | - | IIa |
| **18/226.9** |  | - | II | - | - | II | II | - | - | II | - | II | I | II |  | IIb | - | IIa |
| **18/226.10** |  | II/III | II | II | II | II | II | II | II | II | II | II | I | II |  | IIb | IIa | IIa |
| **18/226.11** |  | - | II | - | - | II | II | II | II | II | - | - | I | II |  | IIb | - | IIa |
| **18/226.10Pla** |  | II/III | II | II | II | II | II | II | II | - | II | II | - | II |  | IIb | IIa | IIa |
| **18/226.11Pla** |  | II/III | II | II | II | II | II | II | II | II | II | II | I | II |  | IIb | IIa | IIa |

^a^I, II or III refers to the archetypal allele from a Type I, II or III, for each molecular marker [33].

^b^Ia=100% homology with GenBank accession no. AF340227 or MT358429 sequences; Ib=T1113C, GenBank accession no. MT361124; IIa=100% homology with GenBank accession no. JX218226 or MT361125 sequences; IIb=G1691T, GenBank accession no. MT361126.

^c^IIa=100% homology with GenBank accession no. AF239285 or MT370491 sequences; IIb= C1013T, GenBank accession no. MT370489.

^d^IIa=100% homology with GenBank accession no. DQ459445 or MT361127 sequences; IIb= C2688T, GenBank accession no. MT361128.
