# Supplementary material for: Isolation and genetic characterization of Toxoplasma gondii in Spanish sheep flocks
Source: Parasit Vectors. 2020 Aug 5;13:396. doi: 10.1186/s13071-020-04275-z (PMC7404076; doi:10.1186/s13071-020-04275-z)
Supplement: Supplementary file 3 — Additional file 3: Table S3. Genotyping allele profile obtained by PCR-RFLP and PCR-sequencing on T. gondii DNA-positive adult sheep myocardium digests. [file 13071_2020_4275_MOESM3_ESM.docx]

**Additional file 3: Table S3.** Genotyping allele profile obtained by PCR-RFLP and PCR-sequencing on *T. gondii* DNA positive adult sheep myocardium digests.

|  |  | **PCR-RFLP alleles^a^** | | | | | | | | | | | | |  | **PCR-Seq alleles** | | |
| --- | --- | --- | --- | --- | --- | --- | --- | --- | --- | --- | --- | --- | --- | --- | --- | --- | --- | --- |
| **ID# Sample** | **Location, year** | **SAG1** | **3’-SAG2** | **5’-SAG2** | **Alt. SAG2** | **SAG3** | **BTUB** | **GRA6** | **c22-8** | **C29-2** | **L358** | **PK1** | **Apico** | **CS3** |  | **SAG3^b^** | **GRA6^c^** | **GRA7^d^** |
| **CC18 G#4** | Plasencia (Cáceres)  2018 | - | - | - | - | - | - | - | - | - | - | - | I | - |  | - | - | - |
| **CC18 G#5** |  | - | - | - | - | - | - | - | - | - | - | - | I | - |  | - | - | - |
| **CC18 G#6** |  | - | - | - | - | - | - | - | - | - | - | - | I | - |  | - | - | - |
| **CC18 G#7** |  | II/III | - | - | - | - | - | - | - | - | - | - | I | - |  | - | - | - |
| **CC18 G#9** |  | II/III | - | - | - | II | II | II | II | - | - | II | I | II |  | IIa | IIa | - |
| **CC18 G#15** |  | - | II | - | - | - | - | - | - | - | - | - | - | - |  | - | - | - |
| **BA18 G#28** | Alburquerque (Badajoz)  2018 | II/III | II | II | II | II | II | II | II | II | - | II | I | II |  | IIb | IIa | - |
| **BA18 G#34** |  | II/III | - | - | - | I | - | - | - | - | II | - | I | - |  | Ia | - | - |
| **BA18 G#35** |  | - | - | - | - | - | - | - | - | - | - | - | I | - |  | - | - | - |
| **BA18 G#39** |  | II/III | II | II | II | II | II | II | II | - | - | - | I | II |  | IIb | IIa | IIa |
| **BA18 G#44** |  | - | - | - | - | - | - | - | - | - | - | - | - | - |  | - | - | - |
| **CU18 G#55** | Sisante (Cuenca)  2018 | - | - | - | - | - | - | - | - | - | - | - | - | - |  | - | - | - |
| **CR18 G#56** | Valdepeñas (Ciudad Real)  2018 | - | - | - | - | - | - | - | - | - | - | - | - | - |  | - | - | - |
| **CR18 G#57** |  | - | - | - | - | - | - | - | - | - | - | - | - | - |  | - | - | - |
| **CR18 G#58** |  | - | - | - | - | - | II | - | - | - | - | - | - | - |  | - | - | - |
| **CR18 G#59** |  | - | - | - | - | - | - | - | - | - | - | - | - | - |  | - | - | - |
| **CR18 G#60** | Puertollano (Ciudad Real)  2018 | II/III | II | II | II | II | II | - | II | II | II | II | I | II |  | - | - | IIa |
| **CR18 G#61** |  | - | - | - | - | - | - | - | - | - | - | - | - | - |  | - | - | - |

^a^I, II or III refers to the archetypal allele from a Type I, II or III, for each molecular marker [33].

^b^Ia=100% homology with GenBank accession no. AF340227 or MT358429 sequences; IIa=100% homology with GenBank accession no. JX218226 or MT361125 sequences; IIb=G1691T, GenBank accession no. MT361126.

^c^IIa=100% homology with GenBank accession no. AF239285 or MT370491 sequences.

^d^IIa=100% homology with GenBank accession no. DQ459445 or MT361127 sequences.
